# Manufacturing processes of peanut (*Arachis hypogaea*) allergen powder-dnfp

**DOI:** 10.3389/falgy.2022.1004056

**Published:** 2022-10-11

**Authors:** Stephanie A. Leonard, Yasushi Ogawa, Paul T. Jedrzejewski, Soheila J. Maleki, Martin D. Chapman, Stephen A. Tilles, George Du Toit, S. Shahzad Mustafa, Brian P. Vickery

**Affiliations:** ^1^ Division of Pediatric Allergy / Immunology, University of California San Diego, Rady Children's Hospital, San Diego, CA, United States; ^2^ Medical Affairs, Aimmune Therapeutics, a Nestlé Health Science company, Brisbane, CA, United States; ^3^ United States Department of Agriculture, Agricultural Research Service, New Orleans, LA, United States; ^4^ President / CEO, InBio, Charlottesville, VA, United States; ^5^ Department of Pediatrics, Guy's and St Thomas’ NHS Foundation Trust, London, United Kingdom; ^6^ Rochester Regional Health, University of Rochester School of Medicine and Dentistry, Rochester, NY, United States; ^7^ Department of Pediatrics, Emory University School of Medicine, Atlanta, GA, United States

**Keywords:** manufacturing, peanut allergy, oral immunotherapy, peanut (*Arachis hypogaea*) allergen powder-dnfp, standardization, drug, food

## Abstract

**Background:**

Important components of drug safety, efficacy, and acceptability involve manufacturing and testing of the drug substance and drug product. Peanut flour sourcing/processing and manufacturing processes may affect final drug product allergen potency and contamination level, possibly impacting drug safety, quality, and efficacy. We describe key steps in the manufacturing processes of peanut (*Arachis hypogaea*) allergen powder-dnfp (PTAH; Palforzia®), a drug used in oral immunotherapy (OIT) for the treatment of peanut allergy.

**Methods:**

Established criteria for source material must be met for manufacturing PTAH drug product. Degree of roasting was determined with a Hunter colorimeter. Protein/allergen content, identity, potency, safety, and quality of each batch of PTAH drug substance were assessed with a combustion analyzer, allergen-specific Western blot (immunoblotting), ELISA, and HPLC. Contaminants (ie, aflatoxin) were measured by UPLC.

**Results:**

Roasting degree beyond “light roast” was associated with variable degrees of protein allergen degradation, or potentially aggregation. Relative potency and amounts of protein allergens showed variability due in part to seasonal/manufacturing variability. Proportion of lots not meeting aflatoxin limits has increased in recent years. Up to 60% of peanut flour source material failed to meet screening selection acceptance criteria for proceeding to drug substance testing, mostly because of failure to meet potency acceptance criteria. Other lots were rejected due to safety (ie, aflatoxin) and quality. Influence of potency variation, within specification parameters, on safety/tolerability observed in trials was considered low, in part due to stringent controls placed at each step of manufacturing.

**Conclusions:**

Extensive variability in allergen potency is a critical issue during immunotherapy, particularly during OIT initial dose escalation and up-dosing, as it may result in lack of efficacy or avoidable adverse allergic reactions. Based on EU and US regulatory requirements, the production of PTAH includes manufacturing controls to ensure drug product safety, potency, and quality. For example, although PTAH contains all peanut allergens, each lot has met strict criteria ensuring consistent allergenic potency of Ara h 1, Ara h 2, and Ara h 6. The rigor of PTAH's manufacturing process ensures reliable dose consistency and stability throughout its shelf life.

## Introduction

A critical component of drug safety, efficacy, and acceptability involves controls across the manufacturing and testing process of a drug substance and drug product (1, 2). A drug substance is an active ingredient intended to provide acceptable pharmacological activity or other direct effect used in the diagnosis, cure, mitigation, treatment, or prevention of disease or to affect the structure or function of the human body (3). A drug product is the finished dosage form that contains the drug substance and may include other active or inactive ingredients (3). In the case of oral immunotherapy (OIT) for food allergies, the use of food as the allergen source is intended to have medical and therapeutic effects, as opposed to food that is intended as nutrition (4, 5).

In recent decades, the application of “Good Manufacturing Practice” (GMP) to “allergen standardization” has emerged as a key regulatory priority and the United States (US) Food and Drug Administration (FDA) has issued multiple “Guidance for Industry” documents outlining Chemistry Manufacturing and Controls Guidance for allergens used for diagnosis or treatment (1, 6). In Europe, similar regulatory guidelines exist (7). GMP refers to standards of production, including the physical facilities, equipment, personnel, and other processes involved with allergen manufacturing (1). Despite adherence to GMP, allergens are highly heterogenous, partly because they are derived from natural sources, but also because manufacturing may involve roasting, grinding, defatting, extraction, clarification, and sterilization (8–11) that change the properties of allergens.

Allergen standardization refers to maintaining consistency within manufacturing processes and analytical capabilities between lots of allergen products and between products from different manufacturers; it is intended to improve both safety and efficacy of allergen immunotherapy (12). This requires the use of rigorously qualified and highly characterized reference standards against which each lot must be measured for potency (ie, allergenic activity) as well as other quality attributes (eg, identity). Although not all allergens used in immunotherapy are standardized, for allergens compounded and administered as immunotherapy by practicing allergists in the US (primarily inhalant or venom allergens *via* subcutaneous injection), the Joint Task Force on Practice Parameters, representing the American Academy of Allergy, Asthma, and Immunology (AAAAI) and the American College of Allergy, Asthma, and Immunology (ACAAI) recommends using standardized allergens when available in the practice parameter on immunotherapy:

“…standardized extracts should be used to prepare allergen immunotherapy treatment sets…The advantage of standardized extracts is that the biologic activity is more consistent, and therefore the risk of an adverse reaction caused by extract potency variability should be diminished.” (8).

Similarly, the European Academy of Allergy and Clinical Immunology (EAACI) guidelines on allergen immunotherapy acknowledge the “…need to limit practice to the use of high-quality, standardized allergen immunotherapy products with good evidence of effectiveness…as many available products are not supported by sufficient evidence of efficacy” (13). Additionally, several publications have been prepared by the EAACI Taskforce on Regulatory Aspects of Allergen Immunotherapy and are part of the EAACI Allergen Immunotherapy Guidelines that pertain to manufacturing and quality of allergen immunotherapy and challenges in implementing the recommendations from EAACI for allergen immunotherapy (2, 14). Comparisons of allergen manufacturing and quality control regulations between the US and European Union (EU) have been reviewed previously (14, 15).

In the early 2000s, OIT emerged as a promising strategy based on small, placebo-controlled studies at academic centers and small, uncontrolled studies conducted by private practitioners (16–18). In 2011, a research retreat was organized and sponsored by an advocacy group called the Food Allergy Initiative (now known as Food Allergy Research and Education) (19). This retreat included a variety of stakeholders, including patient advocates, clinicians, pharmaceutical industry members, and representatives from both the National Institutes of Health and FDA. A consensus was reached that there was a significant unmet need for a standardized OIT approach to food allergy treatment. This led to formation of the Allergen Research Corporation (later renamed Aimmune Therapeutics) (20).

After completing both phase 2 and phase 3 clinical trials (21–23), peanut (*Arachis hypogaea*) allergen powder-dnfp (PTAH; Palforzia®) was approved in 2020 by both the US FDA and the EU European Commission (24, 25). “dnfp” refers to the four-letter suffix extension assigned to PTAH and a naming convention applying to biological products as required by the FDA (26). In the US, PTAH is indicated for the mitigation of allergic reactions, including anaphylaxis, that may occur with accidental exposure to peanut (24, 25). PTAH is approved in the US and EU for use in patients with peanut allergy aged 4 through 17 years and is administered using a standard escalating-dose program (24, 25).

**Figure 1:**
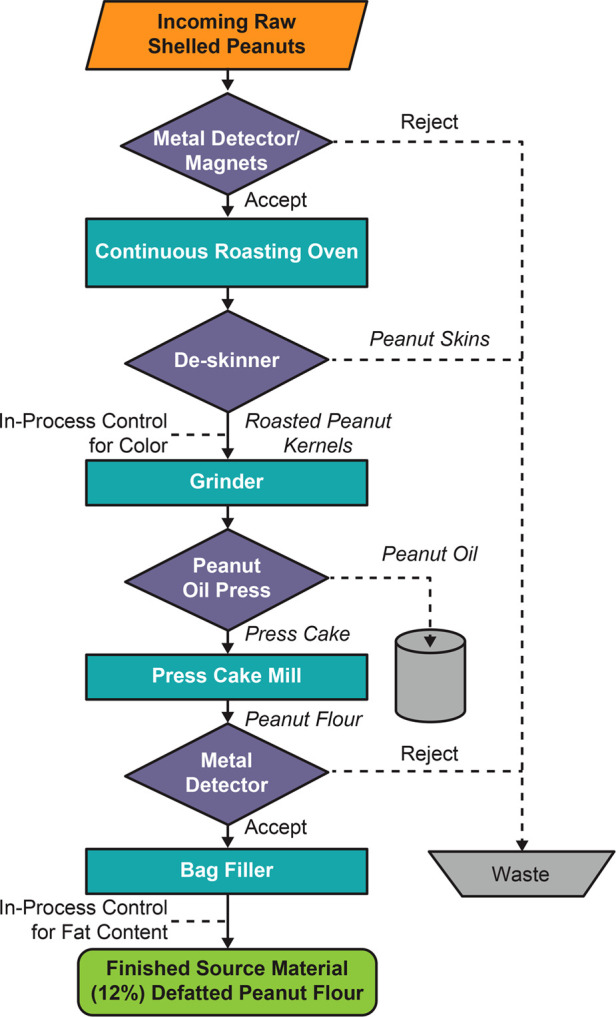
Flowchart of the manufacturing process for peanut flour source material generation at GPTN. Abbreviation: GPTN, Golden Peanut and Tree Nuts.

Source material used for PTAH is a naturally produced material subject to manufacturing processes and storage conditions that impact its use as an approved pharmaceutical product (27, 28). An overview of the multiple quality assurance steps (ie, unit operations and process controls) associated with the manufacturing of source material is shown in Figure 1. The source material for PTAH is 12% defatted, lightly roasted peanut flour produced by the Golden Peanut and Tree Nuts (GPTN) company. GPTN independently selects the raw peanuts for roasting and tests for quality and safety attributes in compliance with food GMP requirements.

These raw peanuts conform to the Code of Federal Regulations Title 7 Part 996, Minimum Quality and Handling Standards for Domestic and Imported Peanuts Marketed in the US (ie, for consumption as food), which limits content of damaged kernels and ensures the peanut stock is minimized for *Aspergillus flavus*, the fungus responsible for aflatoxin (a poisonous carcinogen) contamination in crops (29). The allowable limit of total aflatoxin level in peanut flour distributed as food is 15 parts per billion (ppb) (29). To allow for variations incident to proper grading and handling, a tolerance by weight of 5% split peanut kernels is allowed. Split kernels, due to approximately 50% more surface area per unit mass than the intact kernels, would be exposed to more heat than intact kernels during the roasting process, which can affect allergen quality.

This manuscript will describe the manufacturing of PTAH, the first US FDA and EU European Commission–approved OIT, from the source material of peanut flour to drug substance to the final drug product. We explain the process of peanut source material selection and processing prior to drug substance testing and report the testing and standards for transforming peanut flour material into drug substance.

## Methods

### Manufacturing of palforzia: from source material to drug substance/drug product

To ensure that the peanut flour source material batches designated as drug substance can be used to manufacture PTAH drug product of consistent safety and quality, the batches are subject to a selection process before undergoing formal testing and released as drug substance into GMP production. After receipt and sampling at the testing facility, PTAH drug substance in its container (high-density polyethylene-lined paper bags placed inside a secondary container closure system, a high-density polyethylene drum lined with two low-density polyethylene bags for protection) is stored at 2 °C to 8 °C. Stability is monitored for at least 36 months to ensure lots remain stable within their shelf lives. Drug substance and drug product lots of alternate dosage strengths are selected annually and included in the stability program. Stability of these lots are monitored to ensure the product meets the approved shelf life. Stability monitoring of the final drug product (in capsules and blister packed or in sachets) is also conducted up to 48 months to ensure the potency, safety, and quality of the product remain within specifications until the end of its shelf life.

### Source material

**Figure 2:**
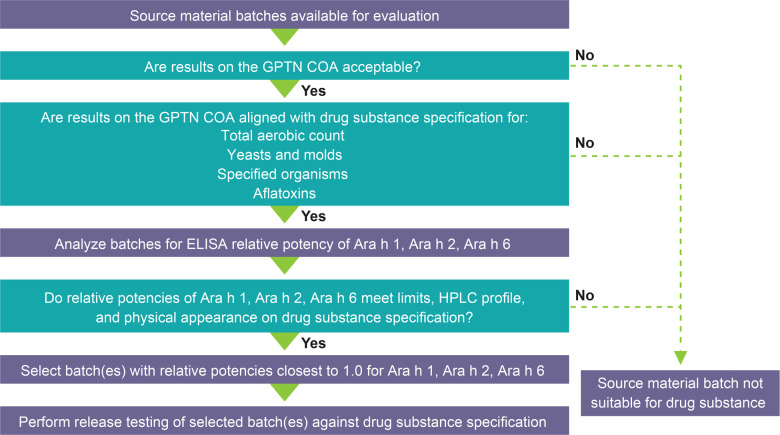
PTAH source material selection process. Abbreviations: COA, Certificate of Analysis; ELISA, enzyme-linked immunosorbent assay; GPTN, Golden Peanut and Tree Nuts; HPLC, high-performance liquid chromatography; PTAH, peanut (*Arachis hypogaea*) allergen powder-dnfp.

The source material selection process for PTAH is rigorous (Figure 2); all clinical study lots are manufactured from 12% fat, lightly roasted peanut flour from GPTN. Source material is stored in the warehouse under ambient conditions until it is shipped for testing and released as drug substance and manufacturing into drug product under pharmaceutical GMPs. The first step in the source material selection process is an evaluation of the results reported on the GPTN certificates of analysis (COA) for the batches being considered. Results on the COA for the source material batch are also evaluated for alignment with acceptance criteria for PTAH drug substance specification for microbiological quality attributes and aflatoxins, as these criteria are more stringent than for GPTN source material.

### Evaluation of bulk peanut flour lots

Evaluation of source material peanut flour lots for pre-selection involves testing for (1) total protein content and quality and relative potency of each of three immunodominant allergens, Ara h 1, Ara h 2, and Ara h 6; (2) aflatoxins, including aflatoxin B_1_ and total aflatoxin; (3) high-performance liquid chromatography (HPLC) relative percent area profile of extracted proteins; and (4) physical appearance, including color, texture, and inherent attributes. Protein content was measured by nitrogen content determined by a Dumatherm nitrogen/protein analyzer (Gerhardt, Königswinter, Germany) using protein/nitrogen conversion factor 5.46. Allergen-specific antibodies custom prepared were used in Western blot and enzyme-linked immunosorbent assay (ELISA) (commercially available and Aimmune developed) methods and validated using internal reference standards. To determine if allergens were intact and confirm immunoassays, product-specific (Aimmune developed) HPLC analysis was used. Briefly, the method used involved a C18 column resin and gradient for elution with ultraviolet detection. The methods have been developed and validated to ensure consistency and robustness in global laboratories. The proportion of screened lots rejected as unsuitable for drug substance was reported.

In the absence of a reference standard specified by regulatory authorities for peanut, the PTAH manufacturing process uses internal reference standards (28). Of note, allergens in currently marketed products are from natural allergen sources and standardization of these products is generally based on internal references and assays (15). PTAH reference standards were prepared from a selected lot of peanut flour that was extensively tested and characterized to establish its potency (27, 28), allergen profile, and quality. The primary reference standard is assigned a nominal potency value of 1.0, stored long term, and used to qualify secondary reference standards, which, in turn, are used in routine lot testing. Additional details on the primary and secondary reference standards are found in the Supplementary Material.

Table 1 lists attributes for the manufacturing process parameters and selected screening tests applied to lots of source material peanut flour being considered for drug substance. In addition to controlling for Ara h 1, Ara h 2, and Ara h 6, other allergens, including Ara h 3 and Ara h 7 through Ara h 17 (which may be considered less clinically relevant yet may be predictive for outcomes such as systemic allergic reactions, epinephrine use, or discontinuation due to gastrointestinal adverse events) were also analyzed. All major and lesser clinically important peanut allergens were identified by peptide mapping and mass spectrometry for identification and relative quantitation. The screening tests include measurements of relative levels of allergens using HPLC and relative potency of immunodominant allergens compared with an internally qualified reference standard using ELISA, protein integrity by HPLC, and levels of aflatoxins using ultra-performance liquid chromatography (UPLC). Color, fat content, moisture, and microbes were analyzed using compendial methods. Other analytic methods were used and have previously been described and reported to regulatory agencies (25, 27, 28).

**Table 1:** Selected tests used during peanut flour source material screening.

Test	Attribute
Relative potency by ELISA^a^	Ara h 1
	Ara h 2
	Ara h 6
Protein integrity and content by HPLC	Area % Ara h 2
	Area % Ara h 6
Aflatoxin by UPLC	Aflatoxin B_1_
	Total aflatoxins
Color	L scale
Fat content	% by weight
Moisture	% by weight
Aerobic plate count	CFU/g
Yeast and mold count	CFU/g

Abbreviations: CFU, colony-forming unit; ELISA, enzyme-linked immunosorbent assay; HPLC, high-performance liquid chromatography; UPLC, ultra-performance liquid chromatography.

^a^ Relative potencies of the peanut allergens are determined by testing against a peanut flour reference standard, which has assigned potency of 1.0 for each allergen. Protein integrity HPLC profile is assessed against the profile of peanut flour reference standard and must be qualitatively comparable. In addition, % peak area of Ara h 2 and Ara h 6 allergens relative to the total peak areas are reported (Figure 4).

### End points and assessments

The identity, potency, and purity of each batch of PTAH drug substance were assessed and confirmed according to the specifications, in accordance with the International Council for Harmonisation (ICH) Q6A and Q6B (30). Source material batches accepted for formal testing as PTAH drug substance were tested according to the drug substance release specification and then released into GMP production of drug product, if acceptable.

Allergen content, aflatoxin, and bioburden (ie, microbial content) of PTAH of source material received from GPTN and the proportion of screened lots rejected as unsuitable for drug substance testing from the years 2018 to 2021 were measured. Correlations of potency with descriptive comparison of clinical findings from clinical trials were conducted, when identified and possible. Correlation and/or associations of relative potency to clinical outcomes were obtained from previously published phase 3 clinical trials of PTAH (22, 23).

## Results

Established critical limits and in-process controls for manufacturing the source material by GPTN must be met to ensure the suitability of peanut flour for drug substance screening and its use in further manufacturing of the allergen source material into the PTAH drug product (Table 2). Additional attributes verified by GPTN (or its contract test laboratories) and documented in the COA include protein content, fat content, moisture, ash, color, aerobic plate count, yeast and mold count, coliform count, *E. coli* count, *E. coli* O157:H7, *Salmonella, L. monocytogenes*, and *S. aureus*.

**Table 2:** Manufacturing process parameters and in-process controls for the allergen source material.

Description	Control Parameters	Purpose
Process Parameters		
Dry roasting conditions	Bed depth	Ensures 5-log reduction in pathogenic organisms (*Salmonella*), controls potency of allergens, protein profile, and physico-chemical attributes of peanut flour derived from the roasted peanut kernels
	Zone 1 temperature	
	Zone 2 temperature	
	Belt speed	
Hydraulic press	Dwell time	Determines the fat content of the source material
In-process Controls		
Color as measured by colorimeter	Ground peanut paste samples	Used to adjust roasting conditions to ensure source material has desired quality
	Source material (peanut flour)	
In-line metal detection	Detection and removal of:	Ensure removal of ferrous and nonferrous metals from the source material
	Ferrous metals	
	Nonferrous metals	
	Stainless steel	
Fat content by near-infrared measurement	Fat content	Used to adjust press dwell time to ensure final product has a desired residual fat content

The actual roasting conditions used to produce peanut flour vary based on the color requirements, the desired taste and aroma profiles of the peanut flour and peanut oil obtained from the roasting process. To ensure the consistent quality of peanut flour used for the manufacturing of Palforzia^®^, only the batches of peanut flour that have been roasted under certain conditions are used. In addition, the color, the presence of metal particles, and the fat content of peanut flour are monitored during the manufacturing of peanut flour from raw shelled peanuts. The peanut flour meeting these requirements is selected for pre-screening, involving testing for attributes summarized in Table 1. Only those peanut flour lots that meet the pre-screen acceptance criteria are then available for extensive testing into drug substance.

### Degree of roasting and color

The impact of degree of roasting on the peanut source material is demonstrated using HPLC testing by peak area proportions of the peanut allergens including Ara h 2, Ara h 6, and Ara h 1 in the elution profiles and is shown in Table 3. The peak areas are tabulated relative to the total peak area of the allergen in light roasted peanut flour. These results suggest that roasting beyond “light roast” affects allergen content, which is shown to be more pronounced for some peanut allergens. In addition, the degree of roasting affects the color (data not shown) of the peanut source material as follows: the light roasted being less roasted is lighter in color, while the dark roast which receives more of the roasting conditions (temperature and time) is darker. Roasting imparts significant chemical processes to peanut and food proteins in general (ie, glycation through the Maillard reaction; protein crosslinking through inter- and intraprotein bonding changes), which contribute the differences in color, flavor, and aroma.

**Table 3:** Source material allergens content in 12% fat peanut flour determined by HPLC in peanut flour manufactured with variable degrees of roasting.

	% Area of Major Allergens^a^			
Type of Source Material	Ara h 2	Ara h 6	Ara h 1	Ara h 3
12% fat peanut flour, light roast (Runner type)	100	100	100	100
12% fat peanut flour, medium roast (Virginia type)	66.5	53.9	59.0^b^	35.6
12% fat peanut flour, dark roast (Runner type)	77.2	76.7	28.6	26.8

HPLC, high-performance liquid chromatography.

^a^ The peak area for each allergen was expressed as the % peak area relative to the total peak area in the HPLC chromatogram and assigned 100% for light roast peanut flour. The % peak areas for each allergen in medium and dark roast peanut flour are shown relative to the peak area of the corresponding allergen in light roast peanut flour.

^b^ Ara h 1 peak elutes as a shoulder in the front side of a large Ara h 3 peak. The Ara h 1 peak is not recognized by the HPLC peak integration software in the medium roast peanut flour due to extensive degradation of the major Ara h 3 peak. Therefore, the Ara h 1 peak area was estimated as roughly 30% of the peak area where normally Ara h 1 elutes.

### Relative potency

**Figure 3:**
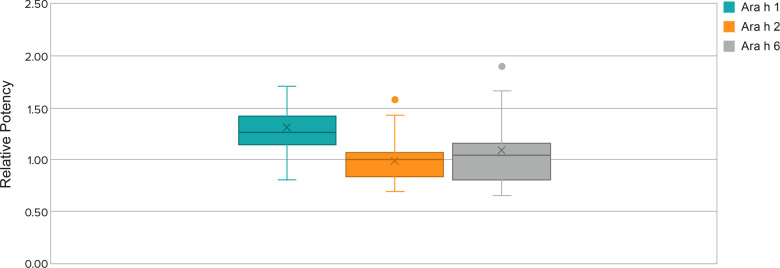
Relative potency ranges for Ara h 1, Ara h 2, and Ara h 6 of selected lots from 2018 to 2021. Whisker plot: The upper and lower whisker bars represent the upper and the lower extreme values. The upper and lower boundaries of the box and the horizontal line represent the upper and lower quartiles and the median. X is the mean. Single data point is an outlier.

The relative potency data (allergen levels) for immunodominant peanut protein allergens varied by about 3-fold within each of the three allergen ELISA tests (Figure 3). Relative potency data among the screened peanut flour lots also show seasonal variability over a span of multiple years (ie, not from one harvest season to the next harvest season). Controlling potency to a tight fold range minimizes the potency variability between each dose-increase step in the multistep dose-escalation treatment. To ensure the consistency of PTAH potency, the range of variation in the allergens is controlled by the peanut flour pre-selection process. The actual range of potency within selected peanut flour lots was even narrower than 3-fold. The presence and consistency of other allergens of lesser clinical importance (Ara h 3 and Ara h 7 through Ara h 17) were also characterized in peanut flour lots.

### Relative amounts of protein allergen

**Figure 4:**
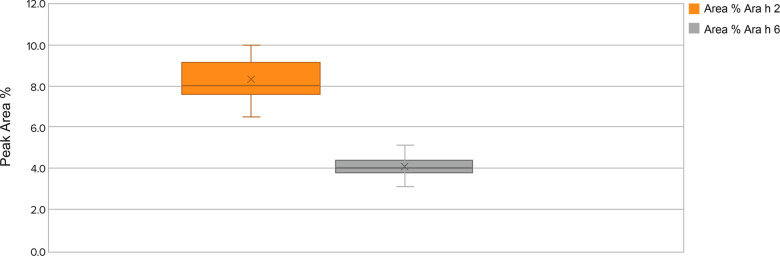
Allergens Ara h 2 and Ara h 6 lot peak area percentage ranges by protein integrity from 2018 to 2021 in source material screened lots. Whisker plot: The upper and lower whisker bars represent the upper and the lower extreme values. The upper and lower boundaries of the box and the horizontal line represent the upper and lower quartiles and the median. X is the mean. Ara h 1 is not displayed due to a very small peak area percentage.

The location, intensity, and peak area measurements by HPLC allow determination of allergen presence, their relative abundance, and intactness (ie, not degraded) within the peanut flour (ie, quality). Levels of allergens within a particular lot and lot-to-lot comparisons with the control standard are also determined by HPLC and ensure consistency. Based on the ranges and integrity of the allergens by HPLC, narrow variation was observed in the relative amount of protein allergens (peak area percentage ranges for allergens Ara h 2 and Ara h 6 are shown in Figure 4).

### Aflatoxin levels

**Figure 5:**
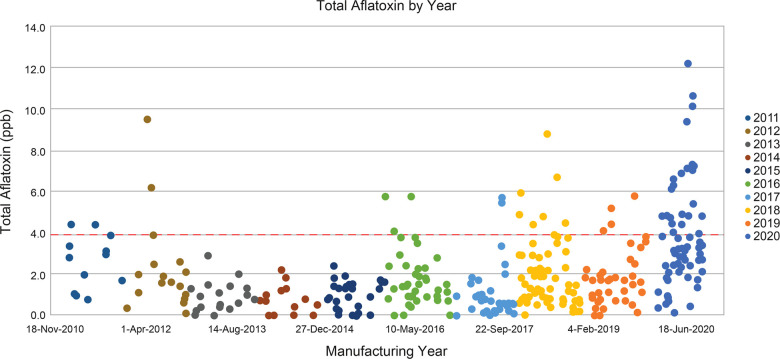
Total aflatoxins in commercial peanut flour lots from 2010 to 2020. Peanut flour lots used in PTAH manufacturing are limited to no more than 4 ppb of total aflatoxins, shown by the red dashed line. GPTN would not manufacture peanut flour from peanut crop where the total aflatoxin exceeds 15 ppb. Abbreviations: GPTN, Golden Peanut and Tree Nuts; ppb, parts per billion; PTAH, peanut (*Arachis hypogaea*) allergen powder-dnfp.

The proportion of the lots not meeting the aflatoxin limits has increased in recent years (2011 to 2020) (Figure 5). A significant proportion of commercial peanut flour lots did not meet aflatoxin total limits for PTAH drug substance use. Total aflatoxin content in commercial peanuts for human consumption is limited to 15 ppb according to the Code of Federal Regulations, Title 7, Part 996.11 (29). Peanut flour lots used in PTAH manufacturing contain substantially stricter limits of total aflatoxins and in particular, aflatoxin subspecies B_1_, to provide a safety margin and to conform to the quality standards required by the European Commission Regulation 1881/2006, Annex 2.1.3 (31). As shown in Figure 5, many of the lots surpass the PTAH manufacturing limit for aflatoxin contamination (4 ppb) and are rejected as source material for PTAH.

### Peanut flour lot rejection overall

Overall, between 2018 and 2021, the majority (up to 60%) of peanut flour source material failed to meet screening selection acceptance criteria for additional drug substance testing (Table 4). The most common reasons for lot rejection included failure to meet relative potency acceptance criteria for one or more immunodominant peanut protein allergens, as well as total aflatoxin level near or higher than the drug substance acceptability threshold. For aflatoxin, rejected lots often differed from the acceptable limit by as much as 3-fold.

**Table 4:** Historical data for peanut flour source material lots screened and rejected from selection for drug substance.

Year	Number of Source Material Lots Selected for Screening	Number of Lots Rejected for Drug Substance Selection	Percentage of Screened Lots Rejected as Unsuitable for Drug Substance Testing
2018	14	8	57%
2019	8	4	50%
2020	7	4	57%
2021	5	3	60%

### Correlation/association with clinical outcomes

The specification range for relative potency by ELISA test for each allergen is approximately 3-fold, which is comparable to the limit of standardized venom or inhalant allergen products (8). The actual relative potency of clinical lots of PTAH was controlled well within the specification limits range. The likelihood of a patient receiving a dose level with a low potency lot followed by an up-dose level with a high potency lot is, therefore, lower than if the allergen had not been standardized and maintained in a specified range. This is especially important for up-dosing where incrementally higher doses are given over time. Control of potency minimizes the risk of a large variability in potency between dose levels, that is, if a PTAH lot at the lower specification limit is used to dose a patient at one dose level and another lot at the upper specification limit is used at the following dose-escalation step.

The PTAH dosing protocol involves stepwise dose increases in peanut protein content that range from 1.2-fold to 2.0-fold in magnitude, except for a 0.5-fold decrease in intended dose between 6 mg at the end of initial dose escalation and 3 mg at the start of the up-dosing phase of treatment. Potency analyses were performed on drug product capsule and sachet lots used in two PTAH phase 3 clinical trials. These assessments represented a total of 5246 participant up-dosing experiences in 520 patients; relative potency ranges and potency ratios of lots used in the PALISADE (ARC003) and ARTEMIS (ARC010) clinical trials are shown in Table 5. For the up-dosing experiences, the frequency distribution of various ratio dose escalations due to both dose and potency in the two clinical studies was analyzed. When the measured potencies of drug product lots were applied to the intended dose increases, 95% of escalations ranged from 0.87 to 2.38, 1.00 to 2.50, and 1.00 to 2.25 for Ara h 1, Ara h 2, and Ara h 6, respectively. Potency increases were based on the actual potencies of the lots used at lower dosing level vs. the next higher dosing level, combined with the increase between the two dosing levels.

**Table 5:** Summary of the relative potency ranges and potency ratios of capsule and sachet lots used in ARC003 and ARC010 clinical studies.

Clinical Study	Number of Drug Product Batches	Ara h 1		Ara h 2		Ara h 6	
		Potency Range^a^	Ratio^b^	Potency Range^a^	Ratio^b^	Potency Range^a^	Ratio^b^
PALISADE^c^ (ARC003)	8	0.68–1.59	2.34	0.72–1.34	1.86	0.63–1.26	2.00
ARTEMIS^d^ (ARC010)	11	0.68–1.25	1.83	0.93–1.34	1.44	0.75–1.26	1.68
Total	19	0.68–1.59	2.34	0.72–1.34	1.86	0.63–1.26	2.00

The data shown in the table are from a total of 5,246 individual up-dosing events for 520 patients.

^a^ Relative to the reference standard.

^b^ Ratio is from highest to lowest potency.

^c^ PALISADE: Peanut Allergy Oral Immunotherapy Study of AR101 for Desensitization (22).

^d^ ARTEMIS: AR101 Trial in Europe Measuring Oral Immunotherapy Success (23).

The up-dosing events of 5246 participants discussed previously included close monitoring in a clinic; adverse events were reported in both PTAH-treated and placebo-treated patients. All but one of these events were graded as either mild or moderate severity. A single severe reaction occurred during an up-dosing visit with 200 mg (after previously taking a 160-mg daily dose). Potency analysis of the lot of drug product used during this visit revealed relative potencies of 1.14, 0.87, and 0.90 for Ara h 1, Ara h 2, and Ara h 6, respectively, which are near the center of the range of the potencies of the lots used. Based on this evaluation, the likelihood that potency variation accounted for the severity of the clinical reaction was considered low. Additional correlation analyses were completed by the FDA and European Medicines Agency during the PTAH approval process (27, 28).

## Discussion

In 1911, Leonard Noon published a report of allergen immunotherapy used for allergic rhinitis caused by grass pollen in the United Kingdom (32). The observation that administering incrementally increasing amounts of an allergen to an allergic person could lead to a “desensitized” state, resulting in symptom improvement, has led to immunotherapy strategies utilizing inhalant allergens, stinging insect venoms, and more recently, foods (33–35).

During the first half of the 20th century, the standard of practice for subcutaneous immunotherapy evolved without regulatory guidance or the benefit of placebo-controlled trials to evaluate safety and efficacy (35, 36). Empiric and anecdotal application of immunotherapeutic principles to treat allergic diseases became widely accepted, but also unintentionally led to routine inclusion of some “allergens” with no efficacy (eg, whole body bee extract), and in some cases, involved potentially unsafe practices (eg, administration of subcutaneous allergen injections to patients with poorly controlled asthma or allowing routine home administration of subcutaneous immunotherapy injections) (37, 38). Safety of allergen immunotherapy might be considered in two ways: involving safety of the drug product itself (ie, protection from harm due to variability in potency or contaminants/impurities) and clinical safety (ie, protection from harm due to biological/physiological effects of the drug when taken by an individual). The nature of drug safety in individuals with peanut allergy is likely heterogeneous; however, ensuring the drug product is high quality and consistent reduces concern that clinical safety is confounded by or due to hazards arising from the drug product.

Adherence to regulations for quality of allergen-specific immunotherapy in Europe and the US is required to obtain marketing approval or authorization (6, 7, 39, 40). Lot-to-lot consistency and shelf-life stability (influenced by stability of individual drug components) are critical to ensure quality. These regulations, therefore, guide the presence of relevant allergens (within specific ranges and including justification for selection), consistency of protein content (within specific ranges), and limits on impurities (14, 39). The preference toward products with proven quality, safety, and efficacy has been demonstrated worldwide over the last 20 years, and requirements have been implemented to distinguish allergen drug products for immunotherapy from non-industrial preparations of allergen immunotherapy directly from food sources that are less controlled and standardized. Yet, challenges arise for analytical characterization of food allergens and correlations between biological potency and protein content in the assessment of quality for allergen immunotherapies (2, 40). Additionally, regulatory guidance appears to be more specific for aeroallergens and insect venom allergies than for food allergen immunotherapy products (40). Use of allergen immunotherapy may be limited by the availability of high-quality, standardized drug products with proven efficacy and safety, as recommended by professional organizations (ie, EAACI, AAAAI, and ACAAI) (8, 13).

Sourcing peanut flour for OIT treatment from GPTN, a food-grade peanut manufacturer, is the starting point for drug substance manufacturing. This peanut flour source material had already undergone substantial analysis and met important quality criteria, yet less than 50% of GPTN lots were suitable for use as drug substance in the PTAH GMP manufacturing process. To ensure the consistency of PTAH potency, the range of variation in the allergens is tightly controlled by the peanut flour pre-selection process. A fundamental requirement for an approved drug is thorough confirmation of drug identity, quality, and safety through all phases of product manufacturing (ie, raw materials, drug substance, in-process, to final drug product), and this is facilitated by adhering to GMP (41). These processes ensure that each packaged dose of drug product meets strict criteria for many attributes throughout its shelf life, including physical, chemical, and immunological properties. Failure to meet acceptance criteria for potency—relative to an in-house reference standard—for each of the immunodominant allergens, Ara h 1, Ara h 2, and Ara h 6, most often accounted for rejection of peanut flour source material, followed by failure to meet acceptance criteria for aflatoxin contamination. In other words, to meet acceptance criteria, peanut flour lots must have appropriate levels of intact immunodominant allergens and low levels of aflatoxin contamination.

A drug substance reference standard is rigorously qualified using all the tests on the drug substance manufacturer's COA, as well as highly characterized using additional analytical methods (US Pharmacopeia or European Pharmacopoeia) that are a necessary activity in the process of drug standardization. As mentioned previously, when preparing allergens for use as subcutaneous immunotherapy treatment, practice guidelines and regulatory authorities suggest choosing standardized allergens when available because of the safety and efficacy advantages of limiting potency variation (8, 12). No such guidelines currently exist for allergens used as food OIT, but the regulatory pathway for the commercial development of an OIT for food allergy has been clarified. Food, when used for medicinal use as a treatment of a food allergy, is considered by regulatory authorities to be a biologic drug, which is regulated in the US by the FDA's Center for Biologics Evaluation and Research (6, 34). The European Pharmacopoeia specifies quality requirements including processes and methods of manufacturing and analysis of medicinal products (39). As such, allergen standardization requires a reference standard(s) as well as a thorough confirmation of its identity, quality, potency, and safety through all phases of product development (12). The reference standard is used to ensure lot-to-lot consistency of allergenic potency. It should be noted that PTAH contains all relevant peanut allergens (as well as the natural mixture of proteins present in peanut, Ara h 1 through Ara h 17) (24), and each lot of PTAH drug substance has met acceptance criteria for relative potency of Ara h 1, Ara h 2, and Ara h 6, each of which is considered immunodominant and clinically relevant; the presence, identity, intact form, and relative levels of these component allergens also met acceptance criteria for consistency relative to both each other and to other lots using reverse-phase HPLC (27, 28).

Extensive variability in allergen potency (eg, if food-grade peanut is used as OIT without further characterization) is particularly important to consider during OIT up-dosing visits when the allergen dose is sometimes intentionally stepped up in 2-fold or more increments. Such variation could present a risk if a lower than intended potency of an allergen component (eg, Ara h 1) would be up-dosed to a higher than intended potency, even when the weight of the peanut flour is appropriate for the intended dose (8). The relevance of this concern is illustrated by the fact that the natural variation in peanut component allergen potency is substantial, even in high-quality, commercially available peanut flours or other peanut-containing products. For example, in one study, the ratio of Ara h 2 to Ara h 1 in a given weight of peanut flour varied more than 40-fold (0.56 to 23.30), depending on the type and source of the peanut flour (9). With a standardized allergen and regulatory body–approved medicine, this kind of potency variation is avoided as evidenced by the consistent relative potencies of Ara h 1, Ara h 2, and Ara h 6 in our analysis of the PTAH lots used in the PTAH phase 3 clinical studies.

A limitation of this analysis is that all specific testing and procedures throughout could not be disclosed due to the proprietary nature of the manufacturing process. However, information has been provided to regulatory authorities to determine if the manufacturing process and final product meet standard criteria for drug approval and distribution.

## Conclusions

Over the years, the source material for peanut flour has shown extensive variation in relative potency, protein component content, and aflatoxin levels. This variability in allergen potency is, in part, due to combined seasonal and peanut flour manufacturing process variations and is an important consideration for a drug used in peanut OIT. This is particularly critical during PTAH up-dosing (dose escalation), as substantial variability has the potential to result in lack of efficacy or trigger an adverse allergic reaction. Rigorous GMP manufacturing process and testing controls have been implemented based on EU and US regulatory requirements to ensure product safety, potency, quality, and safety, at both initial manufacturing, as well as through the end of shelf life of the PTAH drug product. The rigor of PTAH's manufacturing process ensures product consistency between lots and reduces the risk of unintended clinical safety and efficacy outcomes.

## Data Availability

The original contributions presented in the study are included in the article/**Supplementary Materials**, further inquiries can be directed to the corresponding author/s.
